# Non-traumatic Diaphragmatic Eventration With Extrapulmonary Tuberculosis Presenting As Pleural Effusion in an Indian Male: The First Report of Its Type

**DOI:** 10.7759/cureus.58163

**Published:** 2024-04-13

**Authors:** Sankalp Yadav

**Affiliations:** 1 Medicine, Shri Madan Lal Khurana Chest Clinic, New Delhi, IND

**Keywords:** mycobacterium tuberculosis (mtb), thoracentesis, pleural effusion, tuberculosis, non-traumatic diaphragmatic eventration

## Abstract

Tuberculosis is rampant in endemic countries. Extrapulmonary tuberculosis, like pleural effusion, is infrequently reported in outpatient departments. However, diaphragmatic eventration is rare and is not reported in active tuberculosis. Herein, the first-of-its-type case of a diaphragmatic eventration with tuberculous right pleural effusion in an Indian male is presented. The diagnosis was challenging and achieved through radiometric investigations and diagnostic pleural tapping. He was put on an anti-tuberculous treatment based on his weight.

## Introduction

A persistent elevation of the hemidiaphragm without a break in continuity is called diaphragmatic eventration. The pleural and peritoneal layers are not interrupted, the muscle insertions are normal, and the usual orifices are sealed off [1]. With an incidence of 1 in 10,000 live births, it is an infrequent anomaly that could be congenital or due to diaphragmatic palsy in older children and adults. However, in a significant number of cases, it could be idiopathic [2].

Pulmonary and/or extrapulmonary tuberculosis are the results of *Mycobacterium tuberculosis *(MTB) infection [3]. The prevalence of extrapulmonary tuberculosis is 8.4-13.7% [4]. In addition, lymph nodes, pleurae, bones and joints, liver, spleen, meninges, skin, etc. are common sites [3].

Pleural tuberculosis is seldom reported, even in endemic countries [5]. Pleural tuberculosis develops in 3-5% of total tuberculosis patients worldwide, although in high-prevalence situations, this number could rise to 30% [6]. Herein, an exceedingly rare case of left-sided diaphragmatic eventration with extrapulmonary tuberculosis presenting as right-sided pleural effusion in an Indian male is presented.

## Case presentation

A 32-year-old non-diabetic Indian male reported to the outpatient department in the year 2022, with complaints of fever without chills or rigor for 15 days. He also complained of a loss of appetite and about 3 kg of weight loss over two months. Additionally, he also reported periodic dyspnea following food intake, not severe enough to limit daily activities, and he complained of intermittent epigastric discomfort, frequent burps, and water brash for two months. He was consulting local clinicians for these complaints who prescribed paracetamol and antacid for temporary relief. There was no history of cough, night sweat, trauma, or history of tuberculosis in the family or any contacts. He was a working professional in a private company with no history of substance abuse or visits to refugee camps, prisons, or night shelters. He had complaints of epigastric discomfort, frequent burps, and water brash seven years ago when he was diagnosed with left diaphragmatic eventration in the year 2015. He was advised surgery at that time, but he did not follow-up with surgery outpatient department and continued symptomatic treatment.

A general examination revealed a hemodynamically stable male with an ectomorphic build. There was no clubbing, cyanosis, icterus, pallor, edema, or lymphadenopathy. The systemic examination was remarkable for reduced movement on the left hemithorax. Tactile vocal fremitus and breath sounds were also decreased on both sides. Egophony was pronounced in the right lower lobe. There was a reduction in breath sounds in the infrascapular and infra-axillary regions on the left. There were loud crepitations in the entire left lung field. However, respiratory rate was normal and peripheral oxygen saturation was 97% on room air, with no signs indicative of respiratory distress. The rest of the systemic examination was unremarkable.

**Figure 1:**
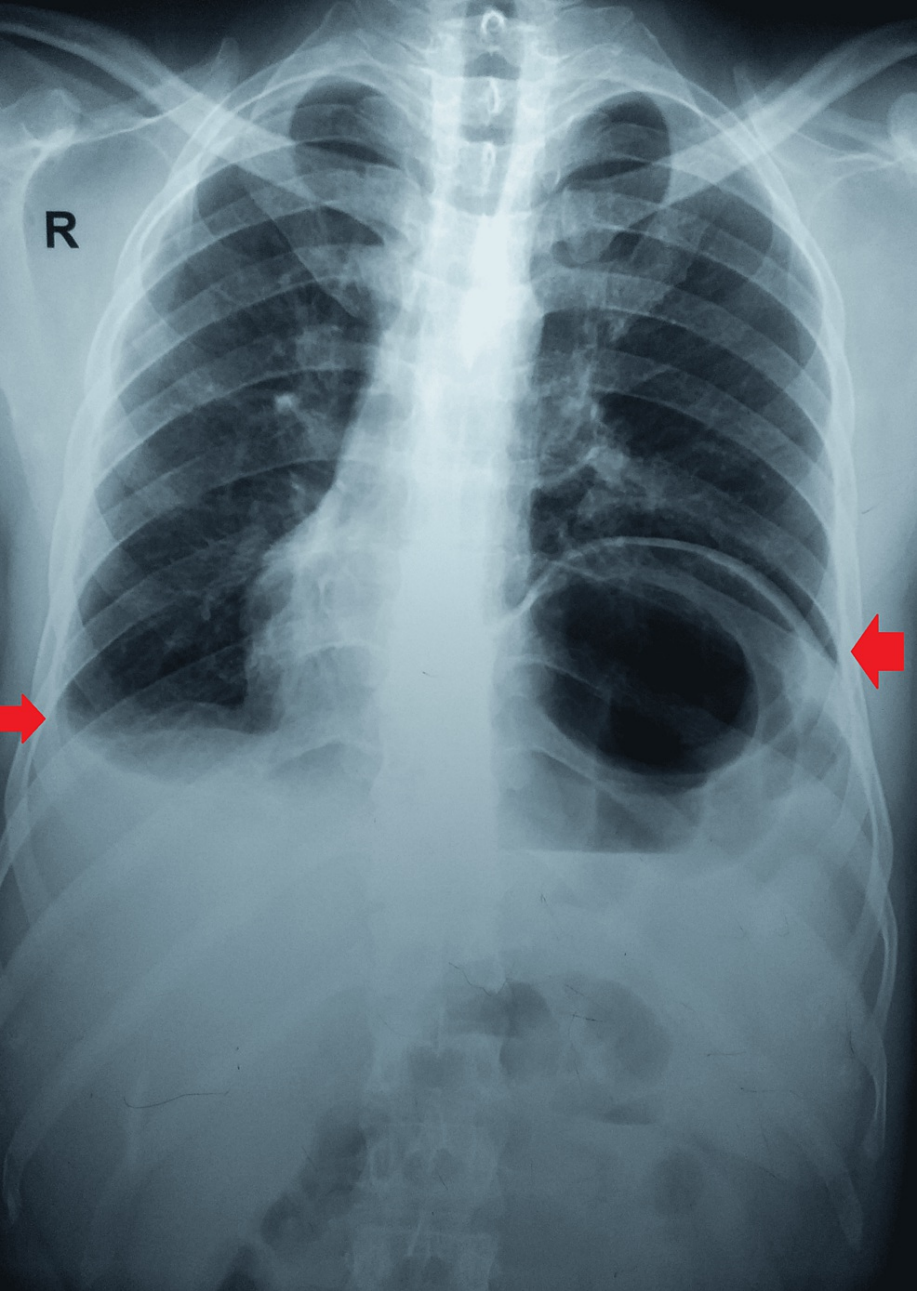
A plain chest radiograph suggestive of a raised dome of the left diaphragm pushing the heart to the right and blunting of the costophrenic angle on the right. Arrows suggestive of left diaphragmatic eventration and right pleural effusion.

The lab data was remarkable for a raised erythrocyte sedimentation rate of 77 mm per hour. His Mantoux test was strongly positive with 23 x 20 mm induration. However, an induced sputum microscopy for the acid-fast bacilli and cartridge-based nucleic acid amplification test (CBNAAT) was negative. A plain chest radiograph was suggestive of a raised dome of the left diaphragm pushing the heart to the right and blunting of the costophrenic angle on the right, suggesting pleural effusion (Figure 1).

**Figure 2:**
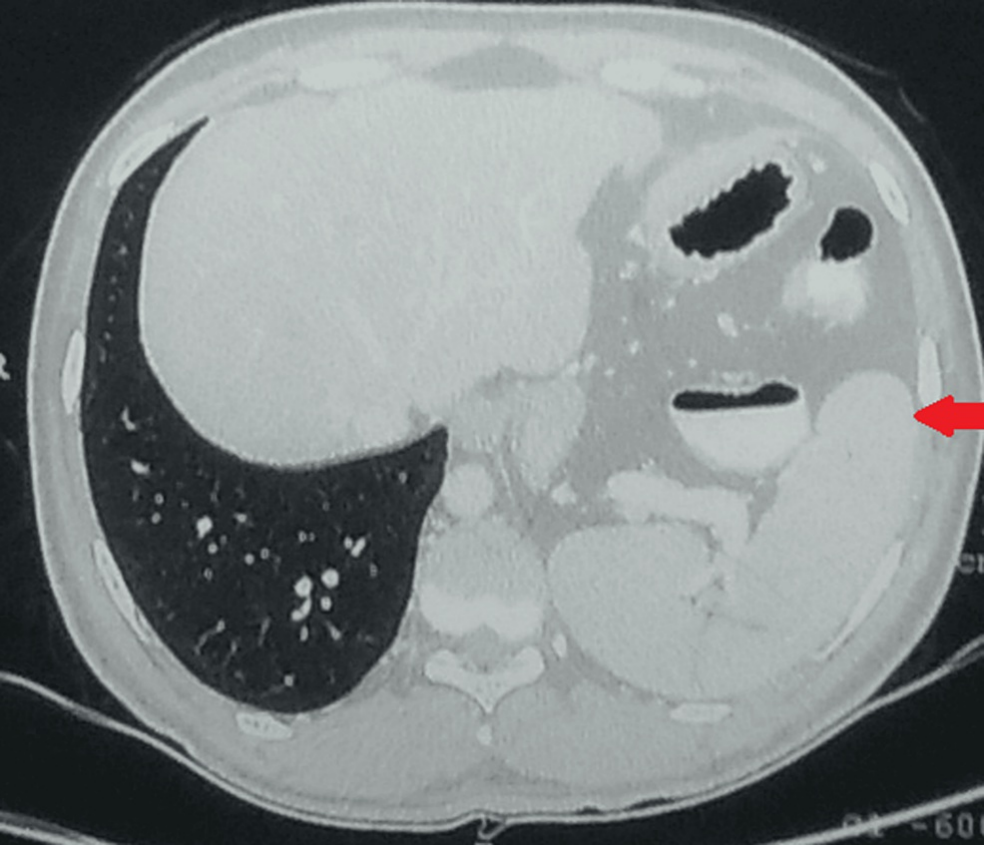
Contrast-enhanced CT of the chest. Arrow suggestive of a raised left hemidiaphragm with a superiorly displaced spleen, stomach, and splenic flexure of the colon, causing a mass effect on the ipsilateral lung, carina, and pushing the heart towards the right.

**Figure 3:**
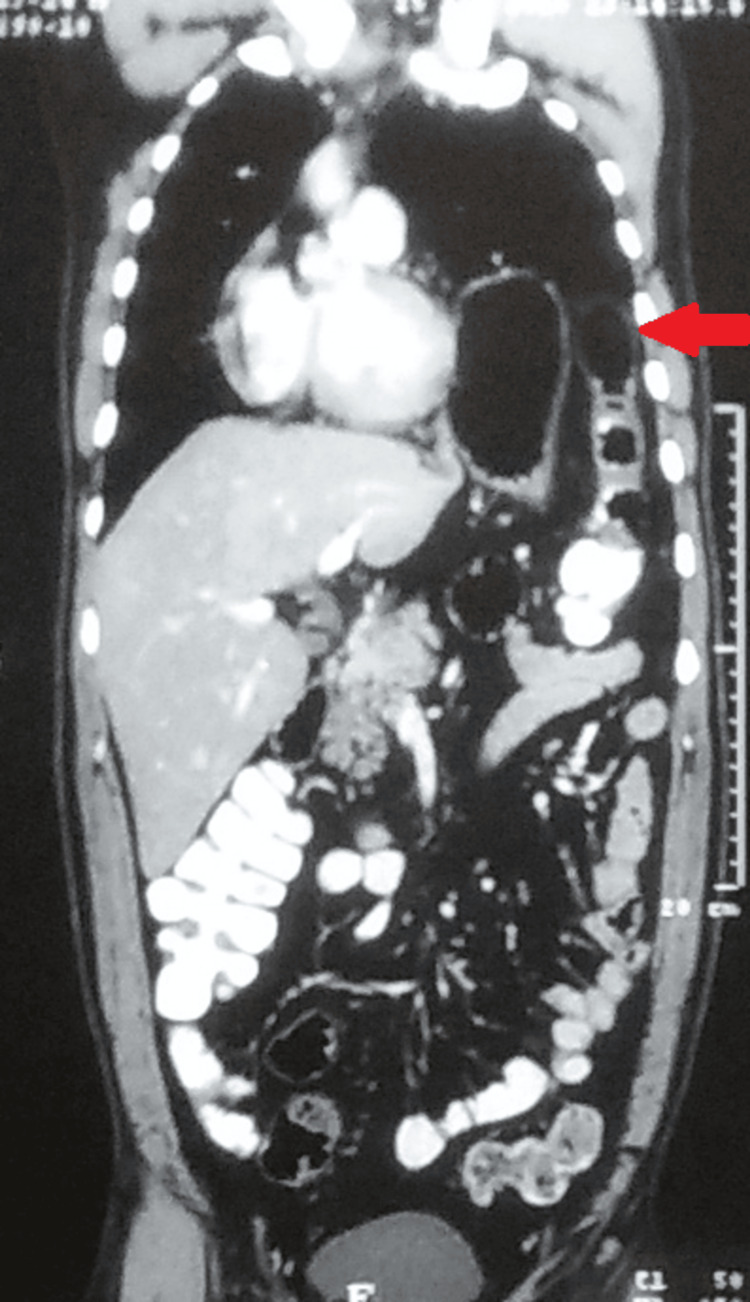
Contrast-enhanced CT of the abdomen. Arrow suggestive of a raised left hemidiaphragm with a superiorly displaced spleen, stomach, and splenic flexure of the colon.

A contrast-enhanced CT of the chest and abdomen done in the year 2015 was suggestive of a raised left hemidiaphragm with a superiorly displaced spleen, stomach, and splenic flexure of the colon, causing a mass effect on the ipsilateral lung, carina, and pushing the heart towards the right (Figures 2,3).

Besides, there was a moderate pleural effusion in the right lung noted on the latest CT of the chest along with findings similar to the previous scan done in 2015. A diagnostic pleural tapping or thoracentesis was done, and the samples were sent for investigations (Table 1).

**Table 1:** Results of thoracentesis ADA: Adenosine deaminase; CBNAAT: Cartridge-based nucleic acid amplification test; MTB: *Mycobacterium tuberculosis*

Test	Result	Reference range
Physical appearance	Straw colored	Colorless
Protein	5.7	1-2 g/dL
Glucose	30 mg/dl	74-106 mg/dL
pH	6.9	7.60-7.64
Cells	85% lymphocytes	75% macrophages
ADA	56.7	<30 U/L
CBNAAT	Negative for MTB	
Culture	Negative	
Pleural fluid protein/serum protein ratio	0.80	
Mesothelial cells	Not seen	

The results were indicative of non-traumatic diaphragmatic eventration on the left hemithorax with extrapulmonary tuberculosis presenting as right pleural effusion. Differentials ruled out were malignancy (no malignant cells on pleural tap), rheumatoid pleurisy (rheumatoid factor negative), empyema (polymorphonuclear neutrophils predominance not seen), and lupus pleuritis (polymorphonuclear neutrophils predominance not seen with antinuclear antibody negative).

**Figure 4:**
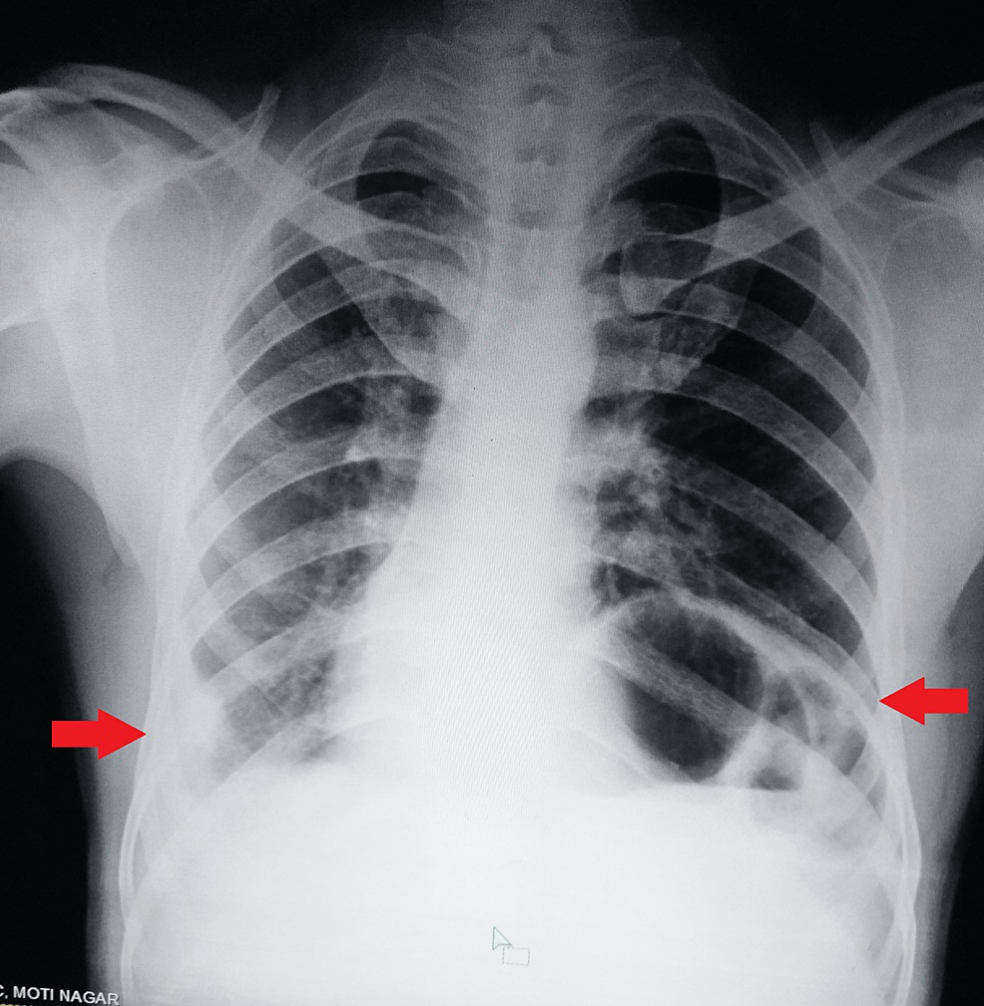
A follow-up chest radiograph showing that there was no progression of disease. Arrows indicate a raised dome of the left diaphragm and blunting of the costophrenic angle on the right or right pleural effusion.

The patient was started on a fixed-dose combination of anti-tubercular drugs, including isoniazid, ethambutol, rifampicin, and pyrazinamide, in the initiation phase for two months, followed by a continuation phase for four months with three drugs. Initially, he responded well with no adverse reactions, gaining weight over the course of a month. However, later, he requested a transfer to his native place, which was granted. He received counselling on treatment adherence, a high-protein diet, and regular follow-up at the nearest infectious diseases clinic. At the patient's three-month follow-up, the condition was clinically stable. In the follow-up chest X-ray, there was no indication of any disease development (Figure 4).

A surgical intervention was not done due to the patient's reluctance and the mild nature of his symptoms, with no flare up of the disease detected during monthly follow-ups.

## Discussion

Diaphragmatic eventration is a very uncommon occurrence [2]. This condition can range in severity from asymptomatic cases that are inadvertently diagnosed to severe dyspnea. Surgical intervention is necessary in cases of severe respiratory distress, recurring infections, and a poor response to conservative treatment. More often reported in men, it affects the left hemi-diaphragm [7]. 

Similar eventration cases in patients with a history of tuberculosis have been documented, and prior reports have suggested a potential connection with tuberculosis [8]. However, a case of active extrapulmonary tuberculosis with a concurrent diaphragmatic eventration as seen in the present case, has never been reported. A few other reports of a diaphragmatic eventration related to enteric fever, polio, influenza, diphtheria, and mumps are also documented [8,9]. Nath et al. reported a case of eventration of the diaphragm with pleural effusion; however, the details of this case are unavailable, and therefore the findings could not be compared [10].

In diagnosing cases like the one presented, imaging techniques play a crucial role. While severe and chronic respiratory distress may necessitate surgical intervention, conservative management suffices for asymptomatic or mildly symptomatic patients, supported by occasional follow-up visits [11]. Diaphragmatic plication, achieved through thoracotomy, laparotomy, or minimally invasive methods, is the preferred surgical procedure aiming to stabilize the diaphragm and optimize lung expansion for maximal inspiration [11]. Surgery is typically indicated for significant elevations causing respiratory distress that impairs daily functioning, although the optimal timing remains a subject of debate, with differing recommendations for observation periods [1].

Modern diagnostic techniques have revealed that tuberculosis-related effusions are typically caused by paucibacillary MTB infections of the pleural space, rather than being solely an immunological phenomenon [11]. Tuberculous pleural effusions often resolve spontaneously but may lead to active tuberculosis in up to two-thirds of cases. Therefore, a high index of suspicion is crucial to diagnosing individuals exhibiting characteristics associated with pleural tuberculosis during the window of opportunity for intervention [6].

As observed in our patient's case, pleural effusions caused by tuberculosis often manifest as moderately sized, predominantly right-sided, and unilateral [6]. Pleural effusions caused by tuberculosis can be challenging to diagnose, especially when lung parenchymal disease is absent on imaging. While pleural fluid culture is the gold standard, it only yields positive results in about 40% of cases [12]. Pleural biopsy offers higher diagnostic rates, but polymerase chain reaction testing sensitivity remains low [6]. The commonly done test is CBNAAT or the Xpert MTB/right iliac fossa (RIF) which is useful in high bacterial load samples, but in paucibacillary cases it has low sensitivity for which Xpert MTB/RIF Ultra is recommended. As the current guidelines in India still use CBNAAT, a negative report on it for detecting MTB was not surprising as seen in the present case. However, it's important to consider tuberculous pleural effusion in patients presenting with a predominantly lymphocyte-based exudative effusion. Determining the etiology of such effusions can be challenging and typically involves a combination of pleural fluid analysis and clinical correlation [5,12]. Adenosine deaminase (ADA) testing in pleural fluid can effectively rule out tuberculosis, especially in low-prevalence settings, and support diagnosis in high-prevalence areas with negative confirmatory tests [6].

In the present case, a nonsurgical strategy backed by anti-tuberculous treatment was chosen because of the patient's infection with MTB* *and the mild nature of the symptoms. Both breathing techniques and counselling for treatment adherence were imparted.

## Conclusions

A case of left-sided diaphragmatic eventration with extrapulmonary tuberculosis presenting as right-sided pleural effusion in an Indian male is presented here. This case stresses the need for reporting such rare presentations. This also emphasizes the need for the dissemination of knowledge about this report so that primary care physicians are aware of such conditions. This will not only help in timely management but also avert any untoward outcomes.
